# Evaluation of Age and Sex-Related Metabolic Changes in Healthy Subjects: An Italian Brain 18F-FDG PET Study

**DOI:** 10.3390/jcm10214932

**Published:** 2021-10-25

**Authors:** Michela Allocca, Flavia Linguanti, Maria Lucia Calcagni, Angelina Cistaro, Valeria Gaudieri, Ugo Paolo Guerra, Silvia Morbelli, Flavio Nobili, Sabina Pappatà, Stelvio Sestini, Duccio Volterrani, Valentina Berti, for the Neurology Study Group of the Italian Association of Nuclear Medicine

**Affiliations:** 1 Nuclear Medicine Unit, Department of Experimental and Clinical Biomedical Sciences “Mario Serio”, University of Florence, 50134 Florence, Italy; flavialinguanti@hotmail.it; 2 Nuclear Medicine Unit, Dipartimento di Diagnostica per Immagini, Radioterapia Oncologica ed Ematologia, Fondazione Policlinico Universitario A. Gemelli IRCCS, 00168 Rome, Italy; marialucia.calcagni@policlinicogemelli.it; 3 Nuclear Medicine Institute, Università Cattolica del Sacro Cuore, 00168 Rome, Italy; 4 Nuclear Medicine, Salus Alliance Medical, 16128 Genoa, Italy; angelinacistaro06@gmail.com; 5 Department of Advanced Biomedical Sciences, University Federico II, 80138 Naples, Italy; valeria.gaudieri@gmail.com; 6 Department of Nuclear Medicine, Poliambulanza Foundation, 25124 Brescia, Italy; upguerra@libero.it; 7 Nuclear Medicine, Department of Health Science (DISSAL), University of Genoa, 16132 Genoa, Italy; silviadaniela.morbelli@hsanmartino.it; 8 Neurology Clinic, IRCCS Ospedale Policlinico San Martino, 16132 Genoa, Italy; flaviomariano.nobili@hsanmartino.it; 9 Department of Neuroscience (DINOGMI), University of Genoa, 16132 Genoa, Italy; 10 Institute of Biostructure and Bioimaging, National Research Council (CNR), 80131 Naples, Italy; sabina.pappata@ibb.cnr.it; 11 Nuclear Medicine Unit, U.S.L. Toscana Centro, Ospedale S. Stefano, 59100 Prato, Italy; stelvio.sestini@uslcentro.toscana.it; 12 Nuclear Medicine Unit, Department of Translational Research and New Technologies in Medicine and Surgery, University Hospital of Pisa, 56126 Pisa, Italy; duccio.volterrani@med.unipi.it

**Keywords:** fluorodeoxyglucose, 18F-FDG, positron-emission tomography, PET imaging, brain, healthy aging, sex differences, menopause, Alzheimer disease

## Abstract

Background: 18F-fluorodeoxyglucose (18F-FDG) positron-emission-tomography (PET) allows detection of cerebral metabolic alterations in neurological diseases vs. normal aging. We assess age- and sex-related brain metabolic changes in healthy subjects, exploring impact of activity normalization methods. Methods: brain scans of Italian Association of Nuclear Medicine normative database (151 subjects, 67 Males, 84 Females, aged 20–84) were selected. Global mean, white matter, and pons activity were explored as normalization reference. We performed voxel-based and ROI analyses using SPM12 and IBM-SPSS software. Results: SPM proved a negative correlation between age and brain glucose metabolism involving frontal lobes, anterior-cingulate and insular cortices bilaterally. Narrower clusters were detected in lateral parietal lobes, precuneus, temporal pole and medial areas bilaterally. Normalizing on pons activity, we found a more significant negative correlation and no positive one. ROIs analysis confirmed SPM results. Moreover, a significant age × sex interaction effect was revealed, with worse metabolic reduction in posterior-cingulate cortices in females than males, especially in post-menopausal age. Conclusions: this study demonstrated an age-related metabolic reduction in frontal lobes and in some parieto-temporal areas more evident in females. Results suggested pons as the most appropriate normalization reference. Knowledge of age- and sex-related cerebral metabolic changes is critical to correctly interpreting brain 18F-FDG PET imaging.

## 1. Introduction

Brain 18F-fluorodeoxyglucose (18F-FDG) positron emission tomography (PET) allows the in-vivo study of regional cerebral glucose metabolism, reflecting neuronal and synaptic activity. Further, 18F-FDG PET has been extensively used to detect metabolic alterations in patients with several neurological diseases vs. healthy aging.

It has been established that normal aging is associated with a progressive decline of synaptic activity, which may affect the cognitive functions [1]. The most common metabolic reductions with advancing age have been observed in the frontal lobes (dorsolateral, orbitofrontal, medial prefrontal, and anterior cingulate cortices), but also in the temporal and parietal lobes bilaterally [2]. Besides, several studies have been conducted evaluating also the effect of sex on cerebral glucose metabolism, with controversial findings. Some authors reported no significant differences [3,4,5]; others found a more pronounced glucose metabolic reduction in frontal areas in males as compared to females, suggesting a major sex susceptibility to age-related decline [6,7,8]. A thorough understanding of the metabolic profile of healthy aging and the effect of sex is essential for an accurate evaluation of metabolic alterations due to neurological disorders, and to ensure a better distinction between normal and pathological brain changes associated with the process of human aging.

Moreover, imaging analysis requires normalization of the radiotracer concentration by scaling the uptake values on a reference region activity to remove inter-individual variations in tracer administered doses and tracer delivery to the brain. Since normalization may have an impact on the detection of reduced regional cerebral glucose metabolism in neurodegenerative diseases, the selection of an appropriate reference has been widely discussed [9,10,11,12,13,14,15,16,17,18,19]. Commonly, the cerebral 18F-FDG uptake was normalized to the averaged whole brain uptake. In some inter-group analyses comparing pathological vs. healthy control patterns, it has been demonstrated that such normalization could underestimate or fail to detect hypometabolic pathological patterns and/or create artifactual hypermetabolism in relatively preserved regions. This might be due to a widespread cerebral glucose hypometabolism related to neurodegenerative pathology [9,13,15,16,17,18,19,20]. Several methods have been proposed to avoid this potential bias, including proportional scaling to the activity of a brain region a priori thought spared by the pathologic process (e.g., white matter, brainstem, cerebellum) [10,12,13,14,17], or with a data-driven process [9,16], generally considered the most accurate method to analyze pathological patterns [21]. However, the impact of the normalization procedure on the detection of the physiological metabolic trend in a healthy aging context was still not clear.

The aim of the present study was to evaluate age-related changes of relative cerebral glucose metabolism in a cohort of cognitively normal subjects, and to assess the effect of sex during aging. Furthermore, the impact on the regional metabolic patterns of normalization method was explored, comparing the cerebral global mean (CGM), the mean of white matter (WM), and of pons activity as reference.

## 2. Materials and Methods

### 2.1. Study Population

In this study, brain PET scans of 151 subjects (84 females and 67 males) aged 20 to 84 included in the normative database of the Italian Association of Nuclear Medicine (AIMN) were analyzed. These subjects were retrospectively selected in 7 centers and defined neurologically normal at the end of a comprehensive neurological work-up. A second verify of the 172 scans initially selected was carried out by an experienced specialist in Nuclear Medicine and 21 patients were excluded from the normative database. There was not a significant difference in age and MMSE score between male and female cohorts (Table 1).

### 2.2. 18F-FDG PET Imaging

The 18F-FDG PET images came from 7 Italian nuclear medicine centers and were obtained using different PET and Computed tomography (PET/CT) integrated scanners (GE Discovery ST, Philips Gemini TF, and GXL, Siemens Biograph). Acquisition scans were performed according to the European and Italian guidelines following standardized procedures [22]. All subjects fasted at least 4 h and had glycemia <140 mg/dL at the time of injection. CT data were used for PET attenuation correction and images were reconstructed with iterative algorithm, following the local routine procedures.

### 2.3. SPM Analysis

The 18F-FDG PET data were analyzed using Statistical Parametric Mapping 12 (SPM). Data were normalized to the SPM probability map template and then smoothed (FWHM 8 mm). Individual global FDG uptake values were normalized by proportional scaling on the CGM, the mean of WM, and pons values.

To investigate the relationship between aging and cerebral glucose metabolism, an SPM correlation analysis was performed setting age as a regressor, and sex, open/close eyes, and scanner as nuisance variables. This analysis has been conducted in the whole group, and after separating male and female subjects into 2 groups. Results were considered significant at *p* < 0.001 with family-wise-error (FWE) correction. The extent threshold was set to 50 voxels. 

### 2.4. ROI Analysis

Regions of interest (ROI) were drawn over all anatomical brain regions and Brodmann Areas (BA) using Pickatlas toolbox; ROIs activity values were extracted and normalized to WM and pons values.

The software used was SPSS (IBM—Statistical Package for Social Science, version 24, Chicago, IL, USA). The correlation between age and 18F-FDG uptake across all ROIs has been evaluated using Pearson correlation coefficient and the relationship was explored by regression analysis. These analyses were performed in the whole group and repeated in male and female groups separately. The results were considered significant at *p* < 0.001. Besides, it was investigated also the age × sex interaction in the entire population and considering *p* < 0.05 as significant.

## 3. Results

### 3.1. SPM Analysis

**Figure 1:**
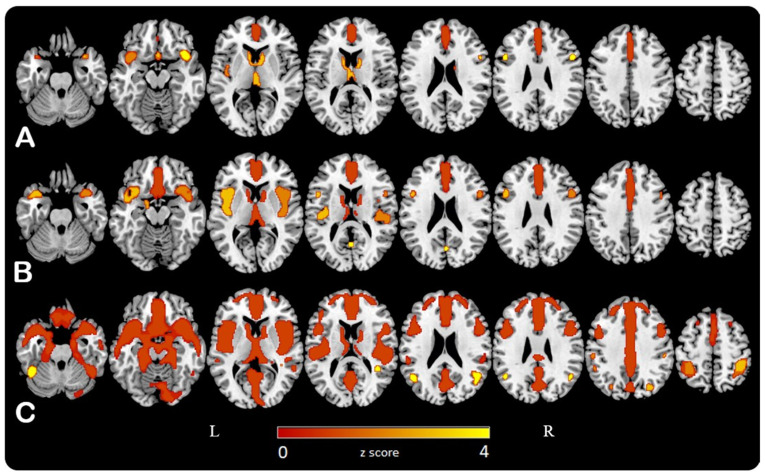
Statistical parametric maps (SPMs) are represented on a color-coded scale (0 < z < 4) and displayed on a standardized brain magnetic resonance imaging. SPMs showed a negative correlation between age and FDG uptake values when cerebral global mean (**A**), white matter (**B**) and pons (**C**) 18F-FDG activity were considered as normalising reference, respectively.

SPM analysis demonstrated a significant negative correlation between age and cerebral glucose metabolism involving mostly frontal lobes bilaterally, especially medial cortices, anterior cingulate cortices, and insula bilaterally. Scaling on pons values, results showed an extension of the negative correlation in lateral parietal areas, precuneus, temporal pole, and medial temporal regions bilaterally (Figure 1). No significant correlation was found in occipital lobes and the primary sensory-motor cortex.

Comparing the three methods of normalization, there was a progressively increased involvement of cerebral parenchyma by the negative correlation, when CGM, WM, and pons were considered as reference, respectively. These patterns were also associated with an increased voxel T-score, with highest values when images were normalizing on pons activity.

**Figure 2:**
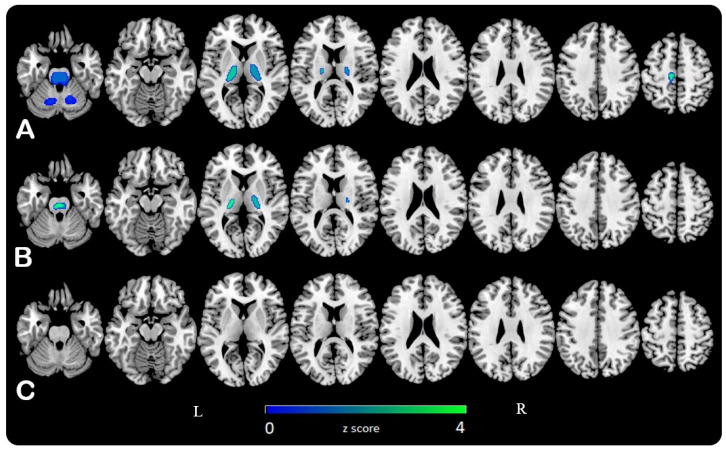
Statistical parametric maps (SPMs) are represented on a color-coded scale (0 < z < 4) and displayed on a standardized brain magnetic resonance imaging. The relative positive correlation between age and FDG uptake in thalami, brainstem and cerebellum when cerebral global mean (**A**) was considered as normalising reference, decreased until is no more evident scaling to white matter (**B**) and pons (**C**) 18F-FDG activity, respectively.

By contrast, the significant relative positive correlation between age and cerebral glucose metabolism in thalami, cerebellum, and brainstem found using CGM as reference, decreased normalizing to WM, and then disappeared using pons values (Figure 2).

**Figure 3:**
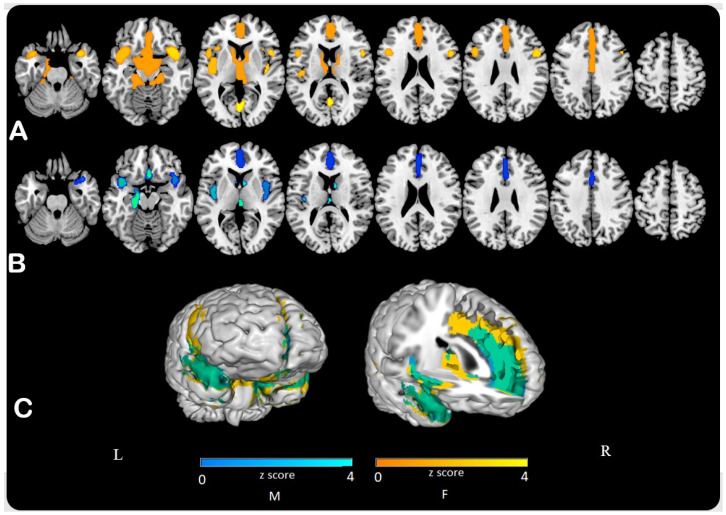
Using P as reference, statistical parametric maps (SPMs) showed a wider negative correlation between age and 18F-FDG uptake value in female (**A**) than in male group (**B**). SPMs are represented on a color-coded scale (0 < z < 4) and displayed on a standardized brain magnetic resonance imaging. In the surface representation (**C**) of SPMs, green represents common areas between females (F) and males (M) analyses.

After analyzing sex cohorts separately using pons as normalizing reference, this pattern of age-related changes was similar in either sex, although with more significant and wider clusters of negative correlation with age in females as compared to males (Figure 3). The frontal lobe was involved both in the male and female groups. In females, there was more extension than in males in the temporal lobes and the posterior cingulate (Table S1).

### 3.2. ROI Analysis

SPSS analysis confirmed previous SPM results, both using anatomical and BA ROIs activity.

When WM was used as reference a significant negative correlation was detected in frontal lobes bilaterally especially in prefrontal medial regions (BA 32–33), the anterior cingulate (BA 24), the insula (BA 13), and orbitofrontal cortices (BA 11–12) but also prefrontal lateral areas (BA 9–10). Using pons values for activity normalization, there was a wider involvement of frontal lobes and extension to more areas of temporal lobes (BA 27–28 and 34–35–36). Moreover, the Pearson coefficient showed more significant results with pons than WM as reference (Table S2).

Normalizing to the WM activity, only pons resulted positively correlated with age, while no positive correlation has been found between age and cerebral glucose metabolism using pons as reference.

Females and males showed the same age-related metabolic pattern with greater involvement of frontal lobe in males and of posterior areas, as posterior cingulate cortices and lingual gyrus, in females (Table 2).

**Figure 4:**
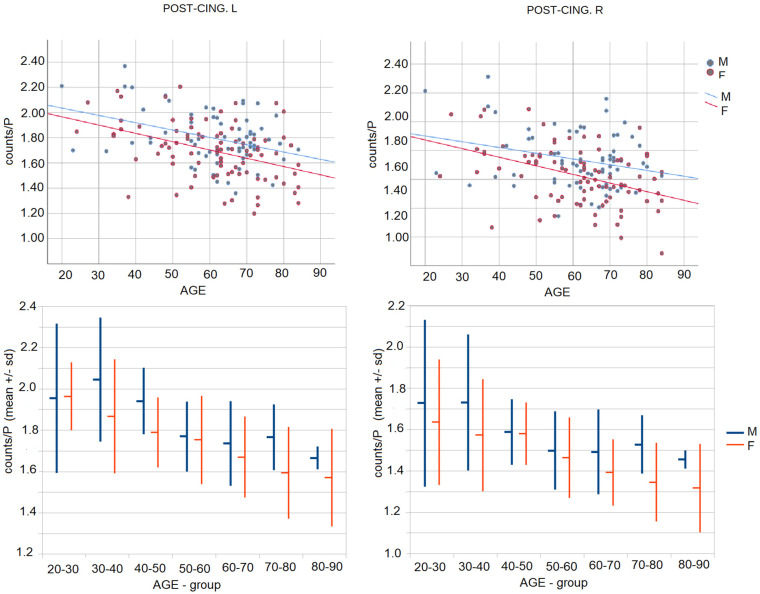
Posterior cingulate (POST-CING) activity in female vs. male, with lower media of 18F-FDG uptake in year decades after 50. M = male, F = female, L = left; R = right.

Analyses demonstrated a statistically significant interaction effect between age and sex on the combined brain activity of anatomical ROIs (F (94, 54) = 1.79, *p* = 0.01, Wilks’ Λ = 0.243, partial η^2^ = 0.757). In particular, after 50 years old females showed a more pronounced reduction in posterior cingulate cortices bilaterally as compared to males (Figure 4).

## 4. Discussion

The current study investigated the effects of aging and sex on regional brain glucose metabolism.

We demonstrated a negative correlation between age and cerebral glucose metabolism involving frontal lobes, especially medial regions, anterior cingulate cortices, insula bilaterally. Lateral parietal areas, precuneus, temporal pole, and medial temporal regions bilaterally, were less affected and their involvement became more evident using pons as reference.

Our findings were consistent with data reported in various neuroimaging studies, describing metabolic reductions with advancing age involving mostly the frontal lobes and anterior cingulate cortices [3,4,5,6,7,8,22,23,24,25,26,27,28,29,30,31,32,33]. This hypometabolic correlation pattern is consistent also with the reported age-associated cognitive decline. Normal aging is characterized by reductions in specific cognitive abilities such as mental speed, executive functions, and episodic memory [34]. Such cognitive deficits, even the memory deficit, have been associated mainly with frontal cortex dysfunction [35,36]. Even the observation of the age-related metabolic reduction in the insula and several regions of temporal and parietal lobes was a consistent finding in the literature [3,4,6,8,24,29,37]. It is interesting to note that these findings were more evident using pons as normalizing reference and in the female group. Scaling on pons values, a different sex susceptibility has probably been pointed out. The only sub-cortical structures involved by the negative correlation were the caudate nucleus bilaterally, also described in literature [4,20,28]. Dopamine plays an important role in several cognitive processes [38,39] and, as known, striatal dopamine functions decline during healthy aging [40,41]. At the same time, the enlargement of ventricles due to age-related atrophy was also known. Consequently, the involvement of a small structure such as caudate nucleus near to ventricles was most probably related to partial volume effect (PVE) due to limited spatial resolution [11,37].

This age-related hypometabolism pattern showed increasing involvement of the brain parenchyma and a more significant correlation (considering both voxel T-score and Pearson coefficient values on SPM and SPSS analyses, respectively) moving from CGM, to WM, to pons activity as normalization reference. Furthermore, the relative hypermetabolism detected in the cerebellum and the brainstem using CGM as a reference was no longer evident scaling to WM and pons values.

Unlike previous studies in literature in which the discussion on the most appropriate reference area for tracer uptake normalization concerned inter-group analyses comparing pathological vs. control patterns [9,10,11,12,13,14,15,16,17,18,19], the present study assessed the impact of the normalization method on the analysis of age-related cerebral glucose metabolism changes in neurologically healthy subjects. Comparing reference areas, proportional scaling to the CGM activity did not turn out the most appropriate normalization method, even if it was the most commonly used, being easy to determine and included in the SPM software. Previous analyses showed the limits of this method of normalization due to the pathological reduction of CGM in patients with neurodegenerative diseases compared to controls [11,15,17,18,19]. A reduction in cerebral glucose metabolism during aging was also known [23,25,28,30]. Consequently, global normalization leads to underestimating the extension of hypometabolic regions and creating an artefactual relative positive correlation in most of the preserved areas. These issues were less evident with WM as reference, but the correct anatomical definition of this region with respect to the cerebral cortex and ventricles should ideally require magnetic resonance imaging (MRI). The use of the core WM might have the advantages of easier anatomical definition using CT co-registered images, less signal spillover from the gray matter. Normalizing for the pons activity it was found a wider and stronger negative correlation between age and cerebral glucose metabolism and no positive correlation in known preserved areas. The pons was a primitive brain region serving as a relay station for the cerebellum and the cerebrum, containing important origin cranial nerve nuclei, with a central role in involuntary functions and respiration. These functions have been preserved during aging. Its preserved glucose metabolism could be derived also from our results, appearing positively correlated with age when CGM was used as reference. Pons is also anatomically easy to define. The small size of the pons may make it vulnerable to random noise, resulting in high variability of the normalization reference value. Anyway, results suggest pons as a good reference area for normalization studying cerebral glucose metabolic aging changes in healthy subjects. 

Regarding differences between the two sex, 18F-FDG PET findings in literature are controversial. Some authors reported no significant sex differences in the age-dependent decline of glucose metabolism in frontal lobes [3,4,5,6,24,26,27]. Besides, Kakimoto and colleagues reported a prevalent reductions in the parietal cortex in men and in the prefrontal cortex, including the Broca area, in women [29]. By contrast, a more severe effect of aging in males in frontal medial cortex [7,8] or in temporal lobes [6] was detected. Even the evaluation of age-related atrophy with magnetic resonance imaging showed heterogeneous results [42,43,44,45,46]. The inconsistency may reflect multiple factors such as sample characteristics, study conditions, normalization methods.

In our study, both males and females were characterized by a similar hypometabolic pattern with a linear reduction of 18F-FDG uptake during aging, more evident in frontal areas. Besides, a significant age by sex effect was detected, related to a worse age-related metabolic reduction in the posterior cingulate cortex bilaterally in women as compared to men after 50 years old. In line with the present study, Hazlett and colleagues [26] found significantly greater age-related cerebral glucose metabolic decline in left cingulate gyrus in women than men. 

Several theories could be advanced to explain sex differences in age-related hypometabolism, such as education, neuropsychological profile, and biological factors, including hormones and genes. It has been demonstrated that age-related brain metabolic reductions could happen in women specifically in the perimenopausal and postmenopausal phase, suggesting an effect of the endocrine asset [47,48,49]. On the other side, in a small sample [50] no sex differences were reported in posterior cingulate, but subsequently, its age-related cerebral glucose metabolic decrease resulted to be prevented by estrogen administration [50,51], supporting an endocrine effect.

Moreover, these findings are consistent with an increased risk of developing Alzheimer’s disease (AD) in women than in men. As known, on 18F-FDG PET, AD is characterized by a specific regional pattern of glucose metabolic reductions in the parieto-temporal cortex and posterior cingulate cortex [33,52]. In particular, a recent study found the posterior cingulate cortex as one of the areas early affected by reduction of cerebral blood flow in the in MCI patients [53]. Neuroimaging studies showed AD-like brain changes in women during the menopause transition characterized by increased brain amyloid-beta (Aβ) deposition, hypometabolism, and neuronal volume loss compared to premenopausal women and men of similar age [48,50,51]. These results are confirmed by a longitudinal study after a 3-year follow up of the same sample [54] indicating the emergence and progression of a female-specific hypometabolic AD-endophenotype during the menopausal transition. The authors suggested that the optimal window of opportunity for therapeutic intervention to prevent or delay the progression of AD endophenotype in women is early in the endocrine aging process.

Some limitations of the present study must be considered. First, a brain atrophy correction of PET images was not performed since MRI on file was not available in the majority of subjects. As known, the most evident change occurred during aging was the enlargement of the ventricles and cortical sulci just in frontobasal and perisylvian structures, while sub-tentorial structures were less affected [45,46]. It has been suggested that the before-mentioned aging pattern of 18F-FDG uptake could be due to PVE, which causes only an apparent metabolic reduction in areas with atrophy [55,56,57]. In contrast, other authors observed a continuing trend of hypo-metabolism in the aging brain, suggesting that the reduction of brain metabolism cannot be accounted for merely by only atrophy [20,25,28,30,58]. The most likely interpretation of our data is that relative hypometabolism in anterior association and limbic areas derives from a combination of atrophy and reduced metabolic values due to synaptic reduction. Second, education and other factors pertaining to cognitive reserve were not considered during analyses while the effect of lifelong cognitive experiences on the aging brain has received consistent evidence and it could influence also the sex differences [5,7]. We could address such topics in future studies.

## 5. Conclusions

Knowledge of age- and sex-related cerebral glucose metabolism changes is critical for accurate detection of metabolic alterations in neurological disorders.

Diminished glucose uptake, reflecting reduced brain metabolism and/or atrophy, correlated with age-associated cognitive decline in this healthy sample.

Considering the results of this study, it would be appropriate to compare the single-patient cerebral FDG-PET to a group of age-related controls. Further, both age and sex should be considered as confounding variables in voxel-based evaluation of neurological disorders.

## Figures and Tables

**Table 1:** Subject characteristics.

	151 Subjects	84 F	67 M	TWO SAMPLE T TEST M-F
AGE (mean ± sd) YEARS	60.78 ± 14.42	60.42 ± 15.21 [80.9% ≥ 50 y.o.]	61.24 ± 13.45 [80.6% ≥ 50 y.o.]	*p* = 0.917
MMSE score (mean ± sd)	29.23 ± 0.94	29.21 ± 1.01	29.29 ± 0.81	*p* = 0.720

MMSE = mini-mental state examination; M = male; F = female; y.o = years old.

**Table 2:** Significant (*p* < 0.001) negative correlation between age and 18F-FDG uptake resulting by SPSS (Statistical Package for Social Science) analyses, using pons 18F-FDG activity as normalization reference in anatomical regions for male (**a**) and female (**b**) separately. ROI = region of interest; r = Pearson coefficient; L = left; R = right.

(**a**)					
**Anatomical ROI Male**	**r**	**Anatomical ROI Male**	**r**	**Anatomical ROI Male**	**r**
insula R	−0.648	FRmed-orb R	−0.524	supMotor area L	−0.457
hipocamp L	−0.617	olfactory L	−0.52	Frsup-medial R	−0.451
cing-ant L	−0.611	paraHipp R	−0.52	temp-pole sup R	−0.448
insula L	−0.601	temp-pole sup L	−0.517	precentral L	−0.446
paraHipp L	−0.593	Frinf-oper L	−0.51	temporal-mid L	−0.445
rectus L	−0.588	heschl R	−0.494	heschl L	−0.441
hippocamp R	−0.577	Rolandic-oper L	−0.494	Frinf-orb L	−0.432
cing-ant R	−0.566	Frinf-oper R	−0.488	temporal sup L	−0.432
olfactory R	−0.546	cing-mid L	−0.484	amigdala R	−0.427
amigdala L	−0.545	parietal-inf R	−0.481	cing-mid R	−0.427
Frsup-medial L	−0.545	Frsup R	−0.478	Rolandi R	−0.424
Frmed-orb L	−0.542	Frinf-orb R	−0.477	Frsup-orb R	−0.419
Rolandi L	−0.532	Frsup-orb L	−0.465	temporal sup R	−0.419
rectus R	−0.531	Frsup L	−0.458	temporal-inf L	−0.414
parietal-inf L	−0.528	Rolandic-oper R	−0.458		
(**b**)					
**Anatomical ROI Female**	**r**	**Anatomical ROI Female**	**r**	**Anatomical ROI Female**	**r**
caudato L	−0.61	FRinf-oper R	−0.484	rectus R	−0.402
insula L	−0.567	FRinf-oper L	−0.481	cing-mid L	−0.395
insula R	−0.559	cing-ant R	−0.446	temp-pole sup L	−0.395
olfactory L	−0.551	FRsup-medial L	−0.446	talamo L	−0.394
paraHipp L	−0.539	cing-mid R	−0.431	parietal-inf L	−0.393
lingual R	−0.528	paraHipp R	−0.429	amigdala L	−0.387
cing-ant L	−0.517	cing-post R	−0.417	FRinf-tri L	−0.387
caudato R	−0.515	rectus L	−0.414	FRmed-orb R	−0.386
heschl L	−0.507	cing-post L	−0.413	parietal-inf R	−0.377
olfactory R	−0.493	FRmed-orb L	−0.41		
heschl R	−0.486	lingual L	−0.41		

## Supplementary Materials

The following are available online at https://www.mdpi.com/article/10.3390/jcm10214932/s1, Table S1: clusters of significant correlation between age and metabolic activity in female (A) and male (M) subjects, resulting from SPM analysis. Table S2: significant (*p* < 0.0001) negative correlation between age and 18F-FDG uptake value resulting by SPSS (Statistical Package for Social Science) analyses, using white matter (A,B) and pons (C,D) 18F-FDG activity as normalization reference in anatomical regions (A,C) and Brodmann areas (B,D).
