# Supplementary material for: Evaluation of Age and Sex-Related Metabolic Changes in Healthy Subjects: An Italian Brain 18F-FDG PET Study
Source: J Clin Med. 2021 Oct 25;10(21):4932. doi: 10.3390/jcm10214932 (PMC8584846; doi:10.3390/jcm10214932)
Supplement: Supplementary file 1 [file jcm-10-04932-s001.zip › jcm-1411184-supplementary.pdf]

Table S1. Clusters of significant correlation between age and metabolic activity in female (A) and male (M) subjects, resulting from SPM analysis.

A.

**Cluster FEMALE**	***p* (FWE-corr) Cluster-level**	**k_E**	***p* (FWE-corr) Voxel-level**	**T**	**x**	**y**	**z**	**Anatomical Location**				
1	0.00E+00	5707	0.00E+00	1.23E+01	−16	−12	−16	L	Limbic Lobe	Parahipp Gyrus	GM	BA 28
			1.36E−11	1.02E+01	0	−16	0	L	Sub-lobar	Thalamus	GM	*
			2.14E−11	1.01E+01	−6	14	2	L	Sub-lobar	Caudate	GM	Caud Body
2	0.00E+00	2124	2.07E−09	8.99E+00	−44	4	−2	L	Sub-lobar	Insula	GM	BA 13
			1.26E−07	8.03E+00	−36	18	−8	L	Sub-lobar	Insula	GM	BA 13
			4.70E−07	7.71E+00	−42	−16	8	L	Sub-lobar	Insula	GM	BA 13
3	0.00E+00	1537	6.95E−08	8.17E+00	40	12	−18	R	Sub-lobar	Extra-Nuclear	GM	BA 13
			1.82E−06	7.39E+00	42	16	0	R	Sub-lobar	Insula	GM	BA 13
			3.93E−06	7.20E+00	36	18	−10	R	Sub-lobar	Insula	GM	BA 13
4	2.93E−09	398	1.92E−05	6.81E+00	6	−92	0	R	Occipital Lobe	Lingual Gyrus	GM	BA 18
			1.11E−04	6.37E+00	0	−74	4	L	Occipital Lobe	Lingual Gyrus	GM	BA 18
			1.52E−04	6.29E+00	0	−64	14	L	Limbic Lobe	Posterior Cingulate	GM	BA 30
5	1.52E−05	75	2.06E−05	6.80E+00	−54	−34	0	L	Temporal Lobe	Mid Temporal Gyr.	GM	*
6	3.92E−05	51	1.39E−04	6.32E+00	−32	−46	48	L	Parietal Lobe	Inf Parietal Lobule	GM	BA 40

B.

**Cluster MALE**	***p* (FWE-corr) Cluster-level**	**k_E**	***p* (FWE-corr) Voxel-level**	**T**	**x**	**y**	**z**	**Anatomical Location**				
**1**	3.80E−08	224	1.76E−06	7.77E+00	−20	−20	−12	L	Limbic Lobe	Parahipp Gyrus	GM	BA 35
			1.32E−05	7.23E+00	−16	−12	−16	L	Limbic Lobe	Parahipp Gyrus	GM	BA 28
			1.02E−04	6.67E+00	−20	0	−18	L	Limbic Lobe	Parahipp Gyrus	GM	BA 34
**2**	5.52E−13	679	2.21E−06	7.71E+00	−40	8	−14	L	Sub-lobar	Extra-Nuclear	GM	BA 13
			2.96E−06	7.63E+00	−42	−6	−4	L	Sub-lobar	Insula	GM	BA 13
			6.93E−05	6.77E+00	−40	−2	8	L	Sub-lobar	Insula	GM	BA 13
**3**	0.00E+00	1177	3.89E−06	7.56E+00	2	40	8	L	Limbic Lobe	Anterior Cingulate	GM	BA 24
			2.08E−05	7.10E+00	2	18	32	L	Limbic Lobe	Cingulate Gyrus	GM	BA 24
**4**	7.90E−14	774	9.12E−06	7.33E+00	40	8	2	R	Sub-lobar	Insula	WM	*
			1.39E−05	7.21E+00	44	−2	−8	R	Sub-lobar	Insula	GM	BA 13
			1.89E−05	7.13E+00	40	−12	2	R	Sub-lobar	Extra-Nuclear	WM	*
**5**	1.06E−05	67	2.06E−05	7.11E+00	2	−24	8	L	Sub-lobar	Thalamus	GM	*
**6**	1.43E−08	257	2.11E−05	7.10E+00	−2	14	−4	L	Sub-lobar	Caudate	GM	CaudHead
		51	1.30E−04	6.60E+00	8	8	8	R	Sub-lobar	Caudate	GM	Caud Body
			3.16E−04	6.35E+00	2	24	−16	R	Limbic Lobe	Anterior Cingulate		BA 24

Table S2. Significant ($p < 0.0001$) negative correlation between age and 18F-FDG uptake value resulting by SPSS (Statistical Package for Social Science) analyses, using white matter (A, B) and pons (C, D) 18F-FDG activity as normalization reference in anatomical regions (A, C) and Brodmann areas (B, D).

A.

**Anat. ROI/WM**	**r**	**Anat. ROI/WM**	**r**	**Anat. ROI/WM**	**r**
insula L	−0.584	paraHipp L	−0.399	cing-mid R	−0.327
insula R	−0.563	FRmed-orb L	−0.395	parietal-inf L	−0.315
cing-ant L	−0.531	rectus R	−0.393	cing-mid L	−0.308
caudato L	−0.516	FRinf-oper R	−0.391	parietal-inf R	−0.301
olfactory L	−0.506	FRsup-medial L	−0.387	rolandic-oper L	−0.292
olfactory R	−0.497	FRmed-orb R	−0.383	FRsup-medial R	−0.287
cing-ant R	−0.460	FRinf-oper L	−0.371	cing-post L	−0.285
heschl R	−0.443	lingual R	−0.362	hippocamp R	−0.284
caudato R	−0.417	paraHipp R	−0.332	lingual L	−0.284
rectus L	−0.415	temp-pole sup L	−0.332	FRinf-tri L	−0.28
heschl L	−0.405	hipocamp L	−0.329		

ROI = region of interest; r = Pearson coefficient; WM = white matter; P = pons; BA = Brodmann area.

B.

**BA ROI/WM**	**r**	**BA ROI/WM**	**r**	**BA ROI/WM**	**r**
BAR13	−0.574	BAR34	−0.401	BAL35	−0.339
BAR25	−0.562	BAR24	−0.399	BAR28	−0.327
BAL25	−0.557	BAR17	−0.398	BAR18	−0.316
BAL13	−0.544	BAR33	−0.397	BAR35	−0.314
BAL34	−0.467	BAL24	−0.396	BAL9	−0.306
BAR32	−0.455	BAL28	−0.395	BAR31	−0.298
BAL32	−0.442	BAL33	−0.378	BAR11	−0.295
BAR41	−0.421	BAL29	−0.376	BAR29	−0.291
BAL27	−0.418	BAL30	−0.365	BAL41	−0.286
BAR27	−0.413	BAR30	−0.357	BAR9	−0.286
BAL23	−0.412	BAL47	−0.355	BAL11	−0.281
BAR23	−0.404	BAR47	−0.340		

ROI = region of interest; r = Pearson coefficient; WM = white matter; P = pons; BA = Brodmann area.

C.

**Anat. ROI/P**	**r**	**Anat. ROI/P**	**r**	**Anat. ROI/P**	**r**
insula R	−0.597	temp-pole sup L	−0.447	FRinf-orb L	−0.342
insula L	−0.579	parietal-inf L	−0.444	FRinf-tri r	−0.341
caudato L	−0.576	FRmed-orb R	−0.443	cing-post R	−0.339
cing-ant L	−0.556	hippocamp R	−0.435	temporal-mid R	−0.337
paraHipp L	−0.554	cing-mid R	−0.428	amigdala R	−0.333
olfactory L	−0.535	cing-mid L	−0.428	FRsup-orb R	−0.327
olfactory R	−0.513	parietal-inf R	−0.418	fusiform L	−0.326
cing-ant R	−0.494	FRinf-tri L	−0.394	supMotor area L	−0.326
FRinf-oper L	−0.493	rolandic-oper L	−0.394	FRinf-orb R	−0.324
rectus L	−0.488	temporal sup L	−0.391	fusiform R	−0.322
heschl R	−0.486	FRsup-medial R	−0.388	precuneo L	−0.316
FRinf-oper R	−0.485	cing-post L	−0.385	FRmid R	−0.311

FRsup-medial L	−0.483	angular R	−0.371	supMarginal L	−0.31
heschl L	−0.475	temporal-mid L	−0.37	talamo L	−0.309
caudato R	−0.473	lingual L	−0.368	temporal-inf L	−0.298
hipocamp L	−0.468	FRsup R	−0.368	precentral L	−0.298
paraHipp R	−0.465	FRsup L	−0.359	supMarginal R	−0.291
FRmed-orb L	−0.465	FRsup-orb L	−0.358	temp-pole sup R	−0.288
rectus R	−0.456	temporal sup R	−0.354	occipital-sup R	−0.282
amigdala L	−0.451	rolandic-oper R	−0.351		
lingual R	−0.447	FRmid L	−0.343		

ROI = region of interest; r = Pearson coefficient; WM = white matter; P = pons; BA = Brodmann area.

D.

**BA ROI/P**	**r**	**BA ROI/P**	**r**	**BA RO/P**	**r**
BAL34	−0.622	BAL47	−0.462	BAL10	−0.37
BAR25	−0.588	BAL29	−0.454	BAL38	−0.359
BAL25	−0.587	BAL33	−0.45	BAR10	−0.355
BAL27	−0.567	BAR47	−0.447	BAL8	−0.339
BAL28	−0.555	BAL30	−0.441	BAR8	−0.337
BAR34	−0.554	BAR18	−0.426	BAR22	−0.332
BAR27	−0.546	BAL9	−0.423	BAL21	−0.331
BAR13	−0.545	BAR30	−0.423	BAL18	−0.328
BAL13	−0.543	BAR31	−0.406	BAR37	−0.326
BAL35	−0.518	BAR9	−0.397	BAL45	−0.325
BAR32	−0.506	BAL44	−0.39	BAL17	−0.319
BAR35	−0.502	BAR36	−0.387	BAR2	−0.318
BAR28	−0.501	BAL36	−0.387	BAR7	−0.318
BAL23	−0.496	BAL11	−0.383	BAR38	−0.315
BAR41	−0.492	BAL31	−0.381	BAR45	−0.315
BAL24	−0.488	BAR11	−0.381	BAR21	−0.3
BAL32	−0.486	BAR44	−0.38	BAL7	−0.297
BAR24	−0.483	BAR29	−0.378	BAR20	−0.287
BAR17	−0.481	BAL41	−0.377	BAL2	−0.285
BAR23	−0.473	BAL40	−0.371	BAR19	−0.28
BAR33	−0.468	BAL22	−0.371	BAL37	−0.28

ROI = region of interest; r = Pearson coefficient; WM = white matter; P = pons; BA = Brodmann area.
